# The compilation of a body weight composite to predict body weight in SA Holstein cattle

**DOI:** 10.1007/s11250-026-05027-4

**Published:** 2026-04-13

**Authors:** D. J. van Niekerk, F. W. C. Neser, M. D. Fair, B. E. Mostert

**Affiliations:** 1 Department of Animal Science, Wildlife and Grassland Sciences, University of the Free State, P O Box 339, Bloemfontein, 9300 South Africa; 2 SA Stud Book, PO Box 270, Bloemfontein, 9300 South Africa

**Keywords:** Age at classification, Days in milk, Linear traits, Total mixed ration

## Abstract

This study reviews the possibility of using body measurements to predict body weight by compiling a body weight composite (BWC) for Holstein cattle in intensive management systems. A data set of 701 records from three farms that feed a total mixed ration was used to build a BWC. The BWC included the following traits and attributes with their proportional contributions: Wither height (18%), body depth (8%), angularity (-16%), rump width (11%), chest width (15%), days in milk at classification (18%) and age at classification (14%). A linear regression was fitted for the BWC against the cows’ realised weights. The regression equation was y = 82.377x + 206.11 with an R^2^ of 0.617. This regression was used in a verification data set to establish the usefulness of the BWC to predict body weight. The correlation between the predicted and the realised weights was 59%, with the average difference between the predicted and realised weights being 3.2%. It is concluded that the BWC is a useful indicator of body weight for Holstein cows in intensive management systems.

## Introduction

In modern dairy management systems body weight recording occurs every time a cow is milked (delaval.com). Unfortunately, the capturing of cow weights still does not occur on a regular basis on many farms. Using linear-type traits, for example rump height (RH), chest width (CW), body depth (BD), rump width (RW) and angularity (ANG), a cow size index can be derived as an indicator of cow weight. This was done by VanRaden et al. (2018) for the US Holstein population. These linear traits were used to define a Body Weight Composite (BWC) that was used in an efficiency index to identify the most efficient cows (VanRaden et al. 2018). Several other studies were conducted where the authors used physical measurements of body traits (e.g. body length, heart girth, height at the rump or the whither) to predict cow weight, for example in in rural areas in Africa to help small-scale farmers estimate the weight of animals for management purposes (Francis et al. 2002; Lukuyu et al. (2016; Comlan et al. (2017; Tebug et al. (2018). These studies measured various body traits (length of body, depth of body, height of the wither, and width of the rump) to predict cow weight.

Dairy herds without the means to weigh cows on a regular basis need a way to determine the size of the cow for management and feeding purposes. One of these management purposes is to identify the most effective and profitable cows. Net income over feed cost is one of the most effective measurements of dairy efficiency and can be defined as the cost of total feed consumed during the period subtracted from the total milk income for the same period (Ribeiro et al. 2008). To determine the cost of feed, the daily feed intake of each cow should be calculated. Unfortunately, no commercial herd in South Africa measures individual feed intake- it is only calculated per group of animals. To be able to calculate feed intake for individual animals, body weight of the animals should be available.

## Aim

To construct and verify a BWC index for South African Holstein herds with intensive management systems using linear type traits and cow weights from three South African Holstein herds that participate in performance recording. This index will be applied to determine a predicted weight for cows without body weight measurements.

## Materials and methods

For this study cow weight and linear type traits from three Holstein herds, located in the Northern Provinces of South Africa, were obtained. According to SA Stud Book (2023) the total number of completed lactations for registered Holstein cows in South Africa, were 4 977. These three herds are milking 1 800 cows, therefore 36% of the national completed lactations for the test year. One of the herds is based in Makhado and is the most northern commercial dairy herd in South Africa. The other two herds are based in Rayton, in Gauteng, and in Davel, in the eastern highveld. All three herds are fed a total mixed ration (TMR) with a base of maize silage. One of the three herds has full-housing for all lactating cows, while the other two herds have full housing for only part of the lactating herd. The herds use predominantly USA semen on their cows and focus in their selection objectives on production, type, fertility and health traits.

Data consisted of linear classification records of 701 first lactation cows, as well as the weight of these cows measured on the day of classification. The assessment was performed by the same classifier in the period May 2020 to July 2021. All weights were obtained from electronic farm systems in which the cows are weighed after every milking. These weights are incorporated into the central database of SA Stud Book (Logix) on a weekly basis. In instances where weights on the evaluation date were not available, weights were obtained from the closest weighing date to the evaluation date, with a maximum range of 7 days before or after the assessment.

The traits that were used in the analysis were:

1. Rump height - The height of the animal is assessed at the rump on a scale from 1 to 9, where 1 is a very short cow and 9 is a very tall cow.
2. Body depth - The depth of the cow’s body is evaluated in the last rib, where 1 is a very shallow cow and 9 is a very deep cow.
3. Chest width - The width of the cow’s chest is assessed on the floor of the chest between the front legs, where 1 is a very narrow chest and 9 is a very wide chest.
4. Rump width – the width of the cow’s rump is assessed by measuring the distance between the pin bones, where 1 is a very narrow setting and 9 is a wide setting.
5. Angularity is evaluated, the wedge form of the cow – side view and top view – as well as the spring of the rib and the openness of the rib, where 1 is a very squire closed rib cow and 9 is a very wedgy open rib cow. Condition contributes to the assessment and an over-conditioned cow will be penalised towards a 1, while a low condition cow will get credited towards a 9 (World Holstein Friesian Federation 2005).

Data analyses were performed using SAS (2017). Pearson’s correlation coefficients were calculated for all traits. A Stepwise regression (SAS 2017) was performed, using a linear regression model that included all traits and attributes that might have an influence on the data. Standardised estimates were obtained for the linear type traits. Data were edited with regards to age at calving and age at classification. All records of animals older than 42 months at calving or at classification were excluded. Furthermore, the classification date should have been later than the calving date, but before the end of the lactation date. This corresponds to the criteria set out in Logix’s editing specifications for the breed’s National Genetic Evaluation (2020 Personal communication SA Stud Book).

For a verification set, weights of 334 animals from the same three herds were obtained, excluding the cows that were used in the first data set. These animals were classified between 29 July 2021 and 30 November 2021. All these animals had on-farm weights for the period 7 days prior to or 7 days after linear assessment. Table 1 gives a summary of the linear data obtained. The BWC, constructed according to the weights in Table 2, was calculated for all cows in the verification data set, to predict the live weight of the cows in the verification data set. This was to determine the applicability of BWC as a predictor of live weight.

**Table 1:** Summary of the Verification dataset for validating BWC as predictor of live weight

HERD 1						
Trait	Number	Min	Max	Average	Median	SD
RH	157	5	9	7.05	7	0.73
CW	157	4	8	6.46	7	0.78
BD	157	5	9	6.89	7	0.72
RW	157	5	9	6.42	6	0.82
ANG	157	6	8	6.60	7	0.52
DIM	157	19	206	113.60	110	52.46
Realised Weight	157	423	868	635	630	92.32
Age	157	711	1182	909.40	891	115.28
BWC	157	2.96	5.80	4.37	4.42	0.55
Predicted Weight	157	450	684	566	571	45.01
Difference*	157	27	-184	-69	-59	-47.31

HERD 2						
Trait	Number	Min	Max	Average	Median	SD
RH	127	5	8	6.72	7	0.60
CW	127	4	7	5.94	6	0.79
BD	127	5	8	6.76	7	0.55
RW	127	5	8	6.13	6	0.72
ANG	127	5	7	6.48	7	0.57
DIM	127	13	204	88.20	88	58.61
Realised Weight	127	415	638	520	514	44.50
Age	127	618	1004	848.45	848	66.25
BWC	127	2.90	5.0	4.01	3.99	0.43
Predicted Weight	127	445	618	536	535	35.61
Difference*	127	30	-20	16	21	-8.89

HERD 3						
Trait	Number	Min	Max	Average	Median	SD
RH	50	6	8	6.72	7	0.57
CW	50	4	8	5.98	6	0.88
BD	50	6	8	6.92	7	0.86
RW	50	4	8	6.74	6	0.86
ANG	50	5	7	6.58	7	0.53
DIM	50	39	320	113.50	125	57.12
Realised Weight	50	451	678	555	550	48.11
Age	50	737	1312	895.36	855	157.39
BWC	50	3.02	6.07	4.23	4.20	0.62
Predicted Weight	50	455	706	555	552	50.83
Difference*	50	4	28	0	2	2.72

ALL HERDS						
Trait	Number	Min	Max	Average	Median	SD
RH	334	5	9	6.88	7	0.68
CW	334	4	8	6.20	6	0.84
BD	334	5	9	6.85	7	0.66
RW	334	4	9	6.28	6	0.80
ANG	334	5	8	6.55	7	0.54
DIM	334	13	320	107.00	102	54.21
Realised Weight	334	415	868	579	564	84.94
Age	334	618	1312	884.42	868	111.56
BWC	334	2.90	6.07	4.21	4.14	0.54
Predicted Weight	334	445	706	553	547	44.74
Difference*	334	30	-162	-6	-17	-40.2

RH = Rump Height, CW = Chest Width, BD = Body Depth, RW = Rump Width, ANG = Angularity, DIM = Days in milk at classification, Weight in kg= Weight at classification, Age in days = Age at classification, BWC = Body Weight Composite*Difference = Predicted Weight – Realised Weight

**Table 2:** Standardised Estimates for the linear traits

Variable	Standardised Estimate	Relative Contribution	Weight in BWC
RH	0.25	17.76%	18%
BD	0.11	8.06%	8%
ANG	-0.22	-15.54%	-16%
RW	0.16	11.57%	11%
CW	0.21	14.75%	15%
dim	0.25	18.21%	18%
age	0.20	14.10%	14%

## Results

The data used in this study are summarised in Table 3.

**Table 3:** Average, standard deviation, median, minimum, and maximum linear classification traits for all three Holstein herds

Herd 1						
Trait	Number	Min	Max	Average	Median	SD
RH	403	5	8	7.02	7	0.60
CW	403	3	8	6.33	7	0.91
BD	403	4	9	6.76	7	0.67
RW	403	4	8	6.53	7	0.79
ANG	403	4	9	6.53	7	0.62
DIM	403	24	348	171.82	170	76.60
Weight	403	416	786	578.84	572	61.78
Age	403	701	1289	896.88	874	117.64

HERD 2						
Trait	Number	Min	Max	Average	Median	SD
RH	240	5	9	6.83	7	0.73
CW	240	3	8	5.84	6	0.87
BD	240	4	9	6.72	7	0.71
RW	240	3	8	6.08	6	0.87
ANG	240	4	8	6.58	7	0.61
DIM	240	5	313	108.00	111	56.02
Weight	240	436	686	536.73	532	49.95
Age	240	657	1250	862.78	854	78.47

HERD 3						
Trait	Number	Min	Max	Average	Median	SD
RH	58	3	9	6.71	7	0.85
CW	58	4	7	5.78	6	0.87
BD	58	5	8	6.55	7	0.72
RW	58	5	7	6.19	6	0.73
ANG	58	5	8	6.53	7	0.56
DIM	58	26	395	104.55	84	70.15
Weight	58	458	630	538.53	537	43.40
Age	58	710	1056	811.88	805	59.83

All Herds						
Trait	Number	Min	Max	Average	Median	SD
RH	701	3	9	6.93	7	0.68
CW	701	4	9	6.12	6	0.93
BD	701	3	8	6.73	7	0.69
RW	701	3	8	6.35	6	0.84
ANG	701	4	9	6.55	7	0.61
DIM	701	5	385	144.44	140	76.66
Weight	701	416	786	560.07	552	59.16
Age	701	657	1289	877.9	858	104.82

RH = rump height, CW = chest width, BD = body depth, RW = rump width, ANG = angularity, DIM = days in milk at classification, Weight in kg = weight at classification, Age in days = age at classification

Most of the animals in the data set were from Herd 1 (403 animals), followed by Herd 2 (240 animals), with the least of the animals from Herd 3 (58 animals). Herd 3 had only one classification event during this period, while Herds 1 and 2 had two events.

The standard deviation for all animals was between 0.61 and 0.93 for linear traits, with CW showing the most variation and ANG the least. No animal received a score of less than 3 for any linear trait, while there were animals that obtained a maximum score of 9 for some of the linear traits, except BD and RW. The maximum classification score for these traits was 8 (Table 3).

The average classification score for animals in Herd 1 was higher for RH (+ 0.19), CW (0.49), and RW (0.31) compared to the other herds. This was also the case for DIM (+ 67.27 days), weight (40.31 kg), and age (34.1 days). The average classification score for all traits was very similar for Herds 2 and 3.

The cows of Herd 1 were on average heavier than those of Herd 2 (12 kg) and 3 (18 kg). Furthermore, the cows of Herd 1 were also on average older at classification compared to the cows of Herd 2 (+ 68 days) and Herd 3 (+ 51 days) (Table 3).

Most of the weight variation in classification was observed in Herd 1, where the cow weight ranged from 416 to 786 kg. This explains the high standard deviation for weight in Herd 1 compared to the other herds. The oldest cow in classification (1 289 days) was found in Herd 1, while the youngest cow in classification (657 days) is in Herd 2. The smallest difference between minimum and maximum scores for linear traits was in Herd 3 for RW (ranging from 5 to 7).

**Fig. 1:**
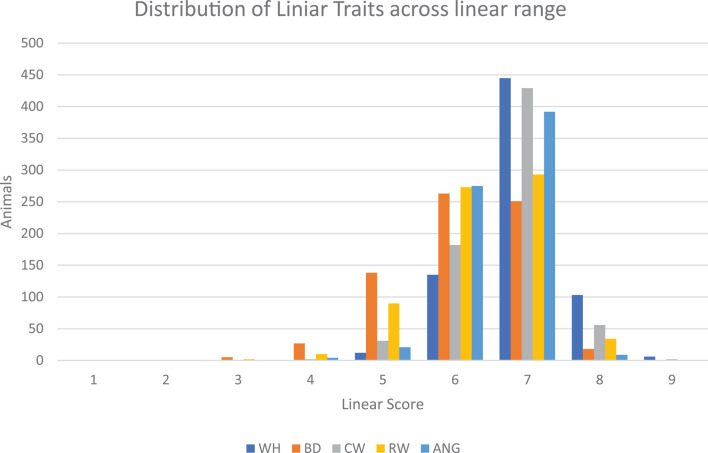
Distribution of the scores of the different linear traits across the range of classification scores over all three herds

Figure 1 indicates the distribution of the scores of the classified animals across the classification range for each linear trait. The highest number of animals obtained a score of 6 or 7 for all traits (WH – 83%, CW – 87%, BD – 73%, RW – 80%, ANG – 95% of all animals).

**Fig. 2:**
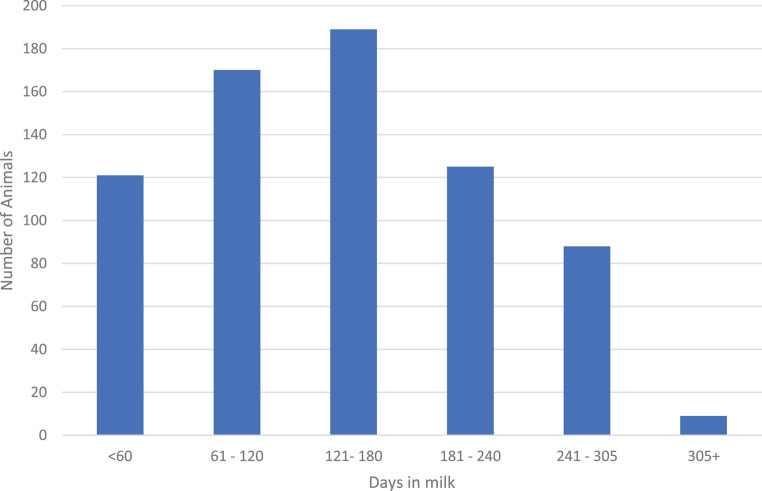
Distribution of classified animals in milk for the three herds

The distribution of animals in days of milk (Fig. 2) indicates that the largest number of animals was classified between 60 days and 240 days in milk. Only nine animals that were classified were longer than 305 days in milk, while 120 animals were classified before they were 60 days in milk.

**Fig. 3:**
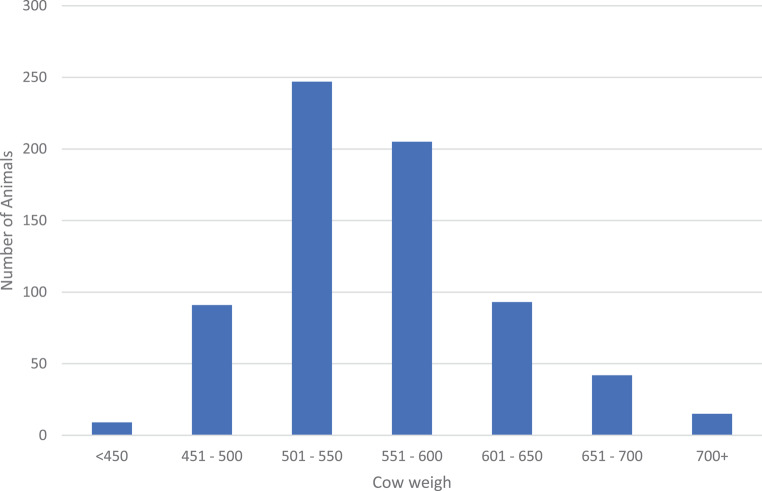
Distribution of the cows over the weight range of measurement in all three herds

The distribution of animals throughout the weight range (Fig. 3) indicates that 64% weighed between 500 and 600 kg on the day of classification. Fifteen animals weighed more than 700 kg and nine animals less than 450 kg.

**Fig. 4:**
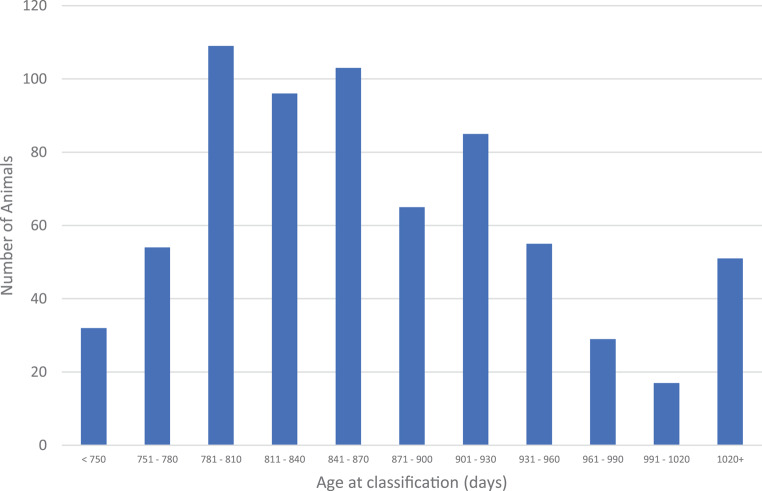
Distribution of the number of animals classified for each month of age at classification

Fifty-one animals were older than 1 020 days at classification (Fig. 4). Most of these (41) were in Herd 1 which is the most northern dairy herd in South Africa, with the distance of travel of the classifier being an issue. Therefore, this herd was not classified according to the schedule, which might cause the animals to be classified at older ages (Personal communication Uys 2021).

**Fig. 5:**
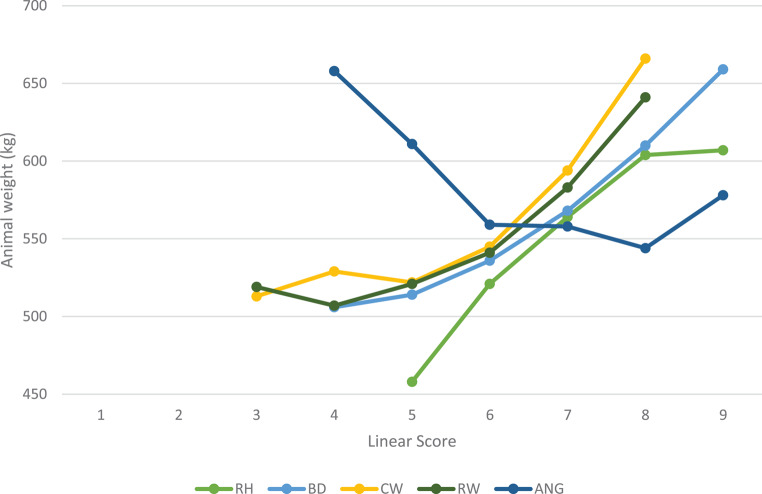
Phenotypic trends of the scores of the different linear traits compared to body weight

From Fig. 5 it can be seen that linear scores increased with an increase in cow weight for all linear traits, except ANG. ANG shows a general decline in weight as the angularity score increases (the animals became wedgier and more open-ribbed).

**Fig. 6:**
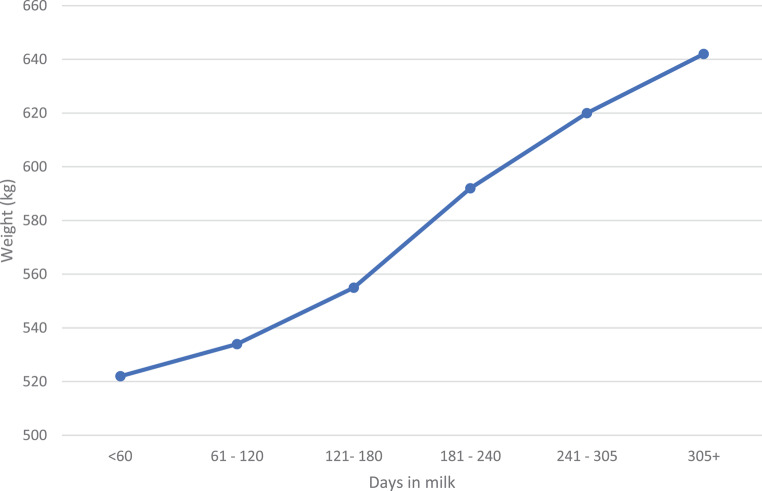
Phenotypic trend of days in milk against body weight

As expected, as days in milk increased (Fig. 6), the weight of the cows also increased due to classifications being done during the cows’ first lactations when they are still in a growing phase.

In Table 4 the Pearson’s correlation coefficients are presented between linear type traits, DIM, age at classification, and cow weight across herds.

**Table 4:** Pearson correlation coefficients between linear type traits, DIM, age at classification with cow weight across herds

Pearson’s correlation	Cow weight	*p*
RH	0.46	< 0.0001
CW	0.53	< 0.0001
BD	0.37	< 0.0001
RW	0.49	< 0.0001
ANG	-0.12	0.0016
DIM	0.57	< 0.0001
Age	0.54	< 0.0001

RH = rump height, CW = chest width, BD = body depth, RW = rump width, ANG = angularity, DIM = days in milk at classification, age = age at classification

A stepwise regression was performed using SAS (2017) to obtain the partial contribution of all independent variables to the dependent variable, body weight, at classification. These independent variables included DIM, CW, RH, RW, ANG, AGE, and BD. The result of the stepwise regression is summarised in Table 5.

**Table 5:** Summary of stepwise regression indicating the significance of DIM, CW, RH, RW, ANG, and BD on cow weight

Step	Variable	Step	Partial *R*^2^	Model *R*^2^	C(*p*)	F-Value	Pr > F
1	DIM	1	0.3196	0.3196	532.421	328.29	< 0.0001
2	CW	2	0.1285	0.4481	302.158	162.58	< 0.0001
3	RH	3	0.0859	0.5340	149.038	128.40	< 0.0001
4	RW	4	0.0260	0.5600	104.044	41.14	< 0.0001
5	ANG	5	0.0240	0.5840	62.6730	40.10	< 0.0001
6	Classification Age	6	0.0245	0.6085	20.3542	43.48	< 0.0001
7	BD	7	0.0085	0.6170	7.0233	15.35	< 0.0001

All variables included in the stepwise regression contributed significantly (*P* < 0.0001) to the variation observed in cow weight. A Model R^2^ of 0.617 was obtained when all variables were included.

Standardised estimates were determined to compare the strength of the effect of DIM, age at classification, CW, RH, RW, ANG, AGE, and BD on cow weight.

To compile the BWC, the results of the standardised estimates were used. In Table 2 the standardised estimates for the linear type traits, as well as their relative contributions with regard to one another, are indicated. These relative contributions were applied as weights for each standardised trait in the BWC compilation.

The BWC was then fitted to the data set to assess its predictability. The average BWC was 4.30 ± 0.54 and the average cow weight 560 ± 59.16 kg (Table 3). The BWC ranged from 2.80 to 6.13.

According to the Pearson’s correlations in Table 4, DIM showed the highest correlation (0.57) with cow weight. As indicated in Fig. 6, cow weight increased with increasing DIM. Similarly, the high correlation (0.54) between age at classification and cow weight confirms that the first lactation cows are still maturing. The highest correlation between linear type traits and cow weight was found to be with CW (0.53), followed by RW (0.49) and RH (0.46). However, there was a low negative correlation between cow weight and ANG (-0.12). This was expected as Fig. 5 indicated that cow weight decreased as the linear score for ANG increased.

In Table 6 it can be seen that the largest changes will occur in days in milk (DIM) and RH (0.25 standard deviation units) with each one standard deviation unit change in body weight, followed by ANG (-0.22 standard deviation units), with the smallest change in BD (0.11 standard deviation units).

**Table 6:** Standardised estimates of DIM, age at classification, CW, RH, RW, ANG, AGE, and BD on cow weight

Parameter Estimates						
Variable	DF	Parameter Estimate	Standard Error	t Value	Pr > |t|	Standardized Estimate
Intercept	1	205.16728	24.13444	8.50	< 0.0001	0
RH	1	21.54412	2.24127	9.61	< 0.0001	0.25
BD	1	9.57191	2.44292	3.92	< 0.0001	0.11
ANG	1	-20.85782	2.52650	-8.26	< 0.0001	-0.22
RW	1	11.37175	1.94640	5.84	< 0.0001	0.16
CW	1	13.08158	1.84287	7.10	< 0.0001	0.21
DIM	1	0.19558	0.02532	7.73	< 0.0001	0.25
Age	1	0.11080	0.01886	5.88	< 0.0001	0.20

**Fig. 7:**
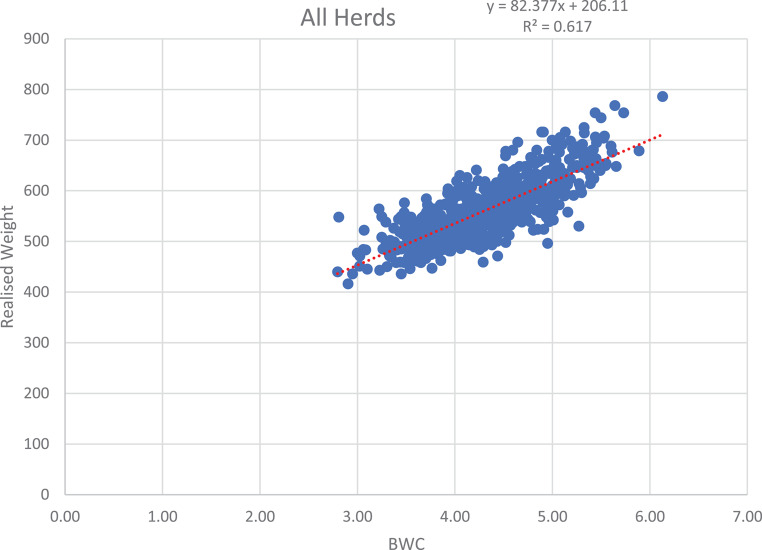
Scatter plot of BWC against realised body weight over all the herds

Figure 7 indicates the relationship between the BWC and realised weight over all herds. A linear regression was fitted that resulted in the regression equation of y = 82.377x + 206.11, where y is body weight and x is BWC, with a R^2^ value of 0.617. Higher order regressions were also fitted, but it did not increase the goodness of fit.

The BWC that is used by US Holstein and SA Stud Book were fitted to the same set of data. Table 7 presents the formula for the BWC used by SA Stud Book and the USA and the BWC constructed in this study. It should, however, be noted that the weightings for the USA and SA Stud Book are applied on breeding values, where DIM and age at calving and classification are adjusted for in the respective Genetic Models.

**Table 7:** Formula for the BWC

Trait	SA Stud Book and USA	This Study
WH	0.23	18%
CW	0.72	15%
BD	0.08	8%
RW	0.17	11%
ANG	-0.47	-16%
DIM		18%
Age		14%

**Fig. 8:**
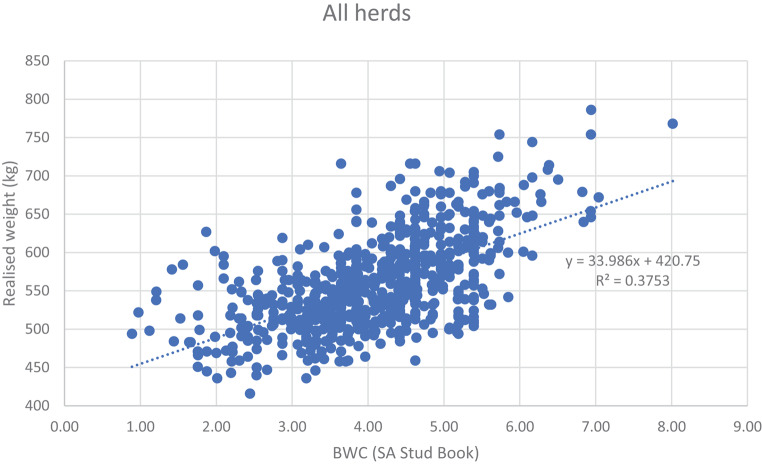
Scatter plot for BWC (SA Stud Book) and the realised weights in kg of the animals

The correlation between the BWC from this study and the BWC used by US Holstein and SA Stud Book applied on this dataset, was 0.78. Figure 8 is the scatter plot for BWC of SA Stud Book & US Holstein and the realised weights of the animals of this study.

Table 1 is a summary of the data from the verification dataset.

The Verification Dataset consisted of 334 first lactation cows from the same three herds that were involved in the original data set (Table 3). Herd 1 contributed most records (161 records), followed by Herd 2 (127 records), with Herd 3 contributing 50 records. The average animal classified between 6.88 and 6.20 for all five linear traits, with an average weight of 576 kg, 107 days in milk and being 884 days old at classification. This correlates with the original data set (Table 3), except for average days in milk that were slightly higher in the original dataset (144 days vs. 107 days). The average BWC on this test-set is 4.04 ± 0.49 (the BWC for the original data set is 4.10 ± 0.54). The average realised body weight based on BWC for the test-set, is 557 kg, which is 3.80% lower than the average body weight (579 kg) obtained from the electronic scales on the farm for the cows.

The trend for the three herds is the same as in the original data set. Herd 1 had larger and heavier animals and were older at classification. Herd 2 had the lowest average DIM (88 days), while that of Herd 1 and 3 were 113 days. Herd 1 had also the highest BWC (4.19 vs. 3.92 and 3.90, for Herds 2 and 3, respectively).

**Fig. 9:**
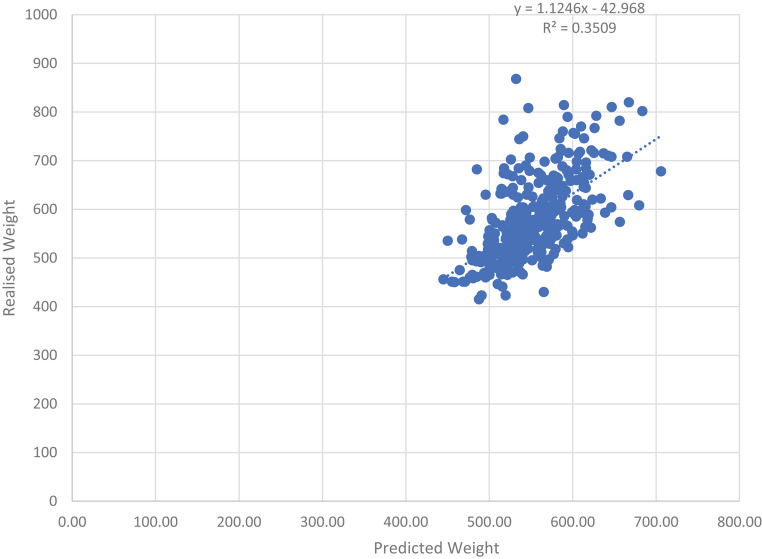
Predicted Weight *versus* Realised Weights across the three herds

The average of the predicted weights for Herd 1, is 10.87% lower compared to that of the realised weights, while Herd 3’s averages are similar. For Herd 2 the average of the predicted weights was slightly higher compared to that of the realised weights (3.08%). Figure 9 indicates the relationship between the predicted weights against the realised weight obtained from the walk-through scale on the day (+-7 days) of classification across the three herds. The correlation obtained between these two weights is 0.59 with a R^2^ = 0.3509.

## Discussion

Studies (Francis et al. 2002; Vallimont et al. 2010; Lukuyu et al. 2016; Comlan et al. 2017; Tebug et al. 2018; Gruber et al. (2018) were done on different populations to determine the correlation between body weight (BW) and body measurements. The majority of the studies used linear assessment to determine the contribution of the trait to the estimate of a body weight. The body measurements that were used were heart girth (HG) or chest width (CW), body length (BL) or angularity (ANG), height at the withers (HW), belly girth (BG) or body depth (DB) and the width of the rump (RW). All these studies found the correlation between HG or CW and BW to be the highest. Francis et al. (2002) studied different cattle breeds in Zimbabwe (Indigenous, Friesian, Brahman, Red Danes and Crossbreds) and found a correlation of *r* = 0.96 between body measurements and live weight. Lukuyu et al. (2016) reported the correlation between BW and body measurements (length of body, heart girth, width of the rump) in crossbred dairy cattle in Kenya to be 0.84. Comlan et al. (2017) studied the correlation in Lagune cattle in Benin and found a correlation of 0.96 between body measurments and live weight. Tebug et al. (2018) also found a correlation of 80% in dairy cattle in Senegal between body measurements and live weight. Gruber et al. (2018) studied the correlation in Fleckvieh, Holstein and Brown Swiss cows in Austria and found a correlation of 82% with both HG and BG and live weight. Vallimont et al. (2010) found the phenotypic correlations between strength (strength is allocated to an animal based on the measurements of the chest, depth and width, with some attention to bone structure) and body weight in Holstein cows in commercial tie-stall barns in Pennsylvania in the USA, to be 0.42. This was the highest correlation found by Vallimont et al. (2010) between different body measurements and body weight.

Vallimont et al. (2010) also found the correlation between body weight and stature to be 0.38, 0.32 with BD, 0.23 with RW and − 0.10 with dairy form.

The findings in this study correlates therefore with the findings of the abovementioned studies, where the highest correlation with BW is found to be with CW (0.53). The negative correlation between BW and ANG in this study (-0.12) corresponds with the finding of Vallimont et al. (2010) of -0.10. This trend is also reflected in the BWC compiled by the US Holstein Society (0.72 on CW and − 0.47 on ANG).

VanRaden et al. (2018) estimated the heritability of BWC for the Holstein population in the US to be 40%. He also determined the phenotypic correlations between BWC and production- and secondary traits. The biggest correlation is with productive life and livability (-0.20) and the lowest is with heifer conception rate (0.02). There is no correlation with daughter pregnancy rate.

In 2018 SA Stud Book implemented a genetic Efficiency Index that includes a BWC index. The genetic increase of BWC over years of birth increased from − 0.5 in 2002 to 0.44 in 2017. Since 2017 it has decreased to 0.3 in 2019, probably due to breeders that started to penalize bulls that breed large body sizes in their breeding objectives.

The aim of the studies done by Lukuyu et al. (2016), Comlan et al. (2017), Tebug et al. (2018) and Gruber et al. (2018) were to determine a model to predict body weight by using body measurements. Lukuyu et al. (2016) used only HG in the prediction of body weight. The model they used were within 95% of the BW. Comlan et al. (2017) used BL, HG and HW in the model and were within 94% of body weight. Tebug et al. (2018) used only HG and the variation from BW for the different breeds varied from − 7.36% to 11.26%. Gruber et al. (2018) used two models. The first model included HG and BG and obtained a coefficient of determination (R^2^) of 83.0. A second model included HG, BG and HW and realised a R^2^ of 83.5. Costigan et al. (2021) used body measurements in Irish Holstein Friesian heifers to determine live body weight. The traits they used, were heart girth, body volume and a polynomial of length, girth and height. The model they compiled correlated very highly with live weight (R^2^ = 0.97). Wangchuk et al. (2017) used four methods to determine body weight from body measurements in Brown Swiss and Jersey cross cattle in Bhutan. All four methods included the measurement of girth and body length. The most accurate method deviated 4.7% and 4.84% from live weight measured by a scale for the two breeds, respectively.

In this study the average difference between realised weights and predicted weights based on the BWC, was − 3.20%, which is in correspondence to the findings of the above studies. The correlation between the realised and predicted weights (0.59) is, however, lower than that obtained in the other studies.

## Conclusion

A Body Weight Composite index was developed from linear type traits, days in milk, and age at classification recorded in three TMR Holstein herds to predict the body weight of first-lactation cows. Validation showed that the index is a useful, though moderately accurate, predictor of cow body weight, with a correlation of 0.59, as applied on the verification dataset. While this accuracy limits its use as a precise phenotypic measure of individual cow body weight, the index enables the exploitation of extensive historical type classification records collected by SA Holstein to derive body weight predictions for cows lacking direct weight measurements. When integrated with milk production data in a genetic evaluation framework, these predicted body weights can be used to derive efficiency-related traits, forming the basis of an Efficiency Index (EI). This allows for the genetic identification of animals that produce higher levels of output relative to body weight, supporting selection for improved biological efficiency, enhanced herd profitability, and the long-term sustainability of dairy breeding programs.

## Data Availability

The datasets generated during and/or analysed during the current study are not publicly available due to agreements signed with SA Stud Book and the SA Holstein Breed Society, but maybe made available from the corresponding author on reasonable request.
